# The baubellum is more developmentally and evolutionarily labile than the baculum

**DOI:** 10.1002/ece3.3634

**Published:** 2017-12-20

**Authors:** Michael Lough‐Stevens, Nicholas G. Schultz, Matthew D. Dean

**Affiliations:** ^1^ Molecular and Computational Biology University of Southern California Los Angeles CA USA

**Keywords:** baculum, baubellum, character mapping, developmental lability, evolutionary lability, sexual dimorphism

## Abstract

Understanding the evolutionary forces that influence sexual dimorphism is a fundamental goal in biology. Here, we focus on one particularly extreme example of sexual dimorphism. Many mammal species possess a bone in their penis called a baculum. The female equivalent of this bone is called the baubellum and occurs in the clitoris, which is developmentally homologous to the male penis. To understand the potential linkage between these two structures, we scored baculum/baubellum presence/absence across 163 species and analyzed their distribution in a phylogenetic framework. The majority of species (*N* = 134) shared the same state in males and females (both baculum and baubellum present or absent). However, the baubellum has experienced significantly more transitions, and more recent transitions, so that the remaining 29 species have a baculum but not a well‐developed baubellum. Even in species where both bones are present, the baubellum shows more ontogenetic variability and harbors more morphological variation than the baculum. Our study demonstrates that the baculum and baubellum are generally correlated across mammals, but that the baubellum is more evolutionarily and developmentally labile than the baculum. The accumulation of more evolutionary transitions, especially losses in the baubellum, as well as noisier developmental patterns, suggests that the baubellum may be nonfunctional, and lost over time.

## 1 INTRODUCTION

Sexual dimorphism, where the same trait takes on different states in the two sexes, is a nearly ubiquitous phenomenon in nature, and understanding the evolutionary forces that lead to sexual dimorphism is an important goal for evolutionary biology (Clutton‐Brock, 2007; Poissant et al., 2010).

The baculum is a highly unusual bone found in the penis—and the baubellum is a bone found in the clitoris—of many mammalian species (Burt, 1960; Layne, 1954). As with many studies of primary sexual traits, the baculum seems to accumulate morphological divergence more rapidly than nonsexual morphologies (Patterson & Thaeler, 1982; Ramm, 2007) consistent with a model of adaptive evolution continuously driving morphological change. The baculum is presumed to be adaptive because of its species‐specific shape (Baryshnikov et al., 2003; Burt, 1936, 1960; Patterson & Thaeler, 1982), rapid evolution under experimental evolution (Simmons & Firman, 2013), and the influence of its shape on male reproductive success (Simmons & Firman, 2013; Stockley et al., 2013).

**Figure 1:**
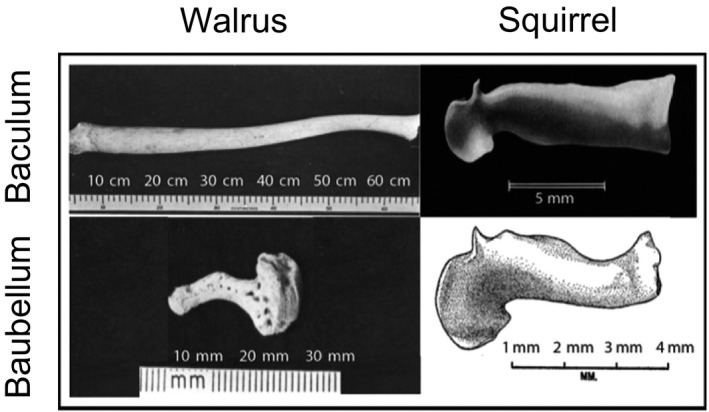
Comparison of walrus/squirrel baculum/baubellum. Note the walrus baculum and baubellum are very different in both size and shape, while the two bones are very similar in the Eastern gray squirrel. *Adapted from Fay, 1982; Burt, 1960; Layne, 1954

The evolutionary and developmental forces affecting the female baubellum, and how they correlate with the baculum, remain poorly understood. In general, the baubellum is much smaller and less morphologically defined than the baculum (Long & Frank, 1968). For example, adult male walruses have the largest known baculum, while adult female walruses have a much smaller and differently shaped baubellum (Figure 1). Other species like Eastern gray squirrels have a baubellum that is similar in both size and shape to the male baculum (Figure 1).

A recent study of approximately 1,000 mammalian species revealed that the male baculum has been gained nine independent times and has been lost 10 independent times (Schultz et al., 2016). These multiple independent transitions provide a unique opportunity to ask if and how evolution and development of the baubellum correlate with the baculum. Here, we analyze the presence/absence of the baubellum across 163 species, and present five main findings. First, the presence/absence of the baculum/baubellum is identical in 134 of the 163 species. Second, in spite of this general correlation, the baubellum showed significantly more evolutionary gains and losses than the baculum, such that states did not match in 29 species. Third, these 29 species are always with a baculum but without a baubellum—we observed no species that lack a baculum but possess a baubellum. Fourth, the baubellum displayed much more variation in development than the baculum, even disappearing with female age in some species. Fifth, the baubellum showed significantly more morphological variation than the baculum. Overall, the baubellum shows more evolutionary and developmental variation than the baculum, indirectly arguing that the baubellum may be relatively nonfunctional.

## 2 MATERIALS AND METHODS

### 2.1 Evolutionary patterns

#### 2.1.1 Scoring baculum and baubellum presence/absence

Presence/absence of the baculum of 1,143 species was taken from table S2 of Schultz et al. (2016). Presence/absence of the baubellum was scored through literature searching and online museum records from August 2015 to January 2017. We were able to find records for 185 species (Table S1). Our primary data came from searches in Google Scholar (https://www.scholar.google.com) and Web of Science (https://webofscience.com/), with the phrases *baubellum*,* baubella*,* os clitoris*,* os clitoridis*,* os glandis*,* ossicle*,* os genital/s*,* os genitale*,* clitoral bone*,* clitoris bone*,* clitorisknochen*,* klitorisknochen*, and *cartilage clitoris*.

We only scored baubella as present if it was (1) shown in photograph or illustration, (2) summarized with measurements, or (3) described in qualitative terms. We scored baubella as absent if it was (1) absent from photographed or illustrated genital dissections, or (2) stated by authors that they were unable to find cartilage or bone upon dissection. Interestingly, many species appear to be polymorphic, in which some but not all females within a species have a baubellum, an issue we specifically address below.

Scoring a baubellum as absent is challenging. The baubellum is generally smaller than the baculum, it is not present in every age class, or remains cartilaginous and difficult to observe in some species (Fay, 1955; Layne, 1952). Nevertheless, we note its absence in one extremely well‐studied model system, the rat. Multiple detailed histological studies have demonstrated that the rat lacks a baubellum (Cherry & Glucksmann, 1968; Glucksmann & Cherry, 1972; Glucksmann, Ooka‐Souda, Miura‐Yasugi, & Mizuno, 1976; Murakami & Mizuno, 1984; Yoshida & Huggins, 1980), even though male rats possess a prominent baculum.

#### 2.1.2 Phylogenetic inference

A large molecular phylogeny of 3,707 mammalian species was taken from supplementary file #1 of Schultz et al. (2016) and was trimmed down to include only species where both the baculum and the baubellum were scored, resulting in 163 species. We then applied stochastic mapping as implemented in the function make.simmap of the R package phytools (Bollback, 2006; Revell, 2012). This is a powerful approach to simulate trait evolution across a phylogenetic tree, while avoiding some of the overly stringent assumptions of a strict parsimony framework. Essentially, character state transitions are distributed across a tree according to an estimated transition rate matrix, with the caveat that each iteration must be consistent with the observed trait states (Huelsenbeck et al., 2003; Nielsen, 2002). This same approach was employed by Schultz et al. (2016) to model baculum evolution. We summarized baculum and baubellum gains and losses from 1,000 iterations of stochastic mapping across each of the four strategies described above, using only the 163 species for which both baculum and baubellum were scored. Visual representations were made using the densitymap function of phytools (Revell, 2012), as well as customized scripts written in R (https://www.r-project.org), available upon request. Branches where a transition occurred in at least 50% of the stochastic mapping iterations were considered “high confidence” transitions.

From the stochastic mapping iterations, we also tested whether transition times differed between baculum and baubellum, using a mixed effects model implemented in the lmer function in the R package lme4 (Bates et al., 2015). Using a likelihood ratio test and a chi‐square distribution with one degree of freedom, we tested whether a model that included bone (baubellum vs. baculum) as a fixed effect explained differences in transition times significantly better than a model that did not. For both models, iteration number was included as a random effect because transition times within an iteration will not be independent from each other.

#### 2.1.3 “Polymorphic” species

Seventeen species were best classified as “polymorphic,” where some females had a baubellum while others of the same age class did not, for example in domestic dogs (Kutzler et al., 2012). We implemented four different strategies of stochastic mapping to account for alternative views of the polymorphic state. First, “polymorphic” was considered a third state in addition to “present” or “absent.” Second, all polymorphic species were assigned the state of “present,” which could be interpreted as a trait state that normally develops but is incompletely penetrant or difficult to observe and occasionally overlooked in the literature. Third, all polymorphic species were assigned the state of “absent,” which could be interpreted as a trait state that normally does not develop. For these second and third models, it is interesting to note that female rats, ferrets, and dogs all develop baubella with additional administration of testosterone (Aucélio et al., 1982; Baum et al., 1982; Glucksmann & Cherry, 1972; Murakami & Mizuno, 1984; Yoshida & Huggins, 1980; Zimbelman & Lauderdale, 1973), and it is possible that variation in hormonal profile explains polymorphism. Lastly, “polymorphic” species were randomly assigned “present” or “absent,” which is some combination of the second and third strategies. For the remainder of this manuscript, these four strategies are referred to as “polymorphic,” “present,” “absent,” and “random,” respectively. All four strategies give qualitatively the same answers (see below).

### 2.2 Developmental patterns

#### 2.2.1 Comparing the development of baubella with bacula

During our literature search, we uncovered five species where multiple males and females from multiple age classes were assessed for the presence of both a baculum and baubellum (Baitchman & Kollias, 2000; Callery, 1951; Fay, 1955, 1982; Friley, 1949; Hawkins et al., 2002; Lauhachinda, 1978; Lönnberg, 1902; Mansfield, 1958; Scheffer, 1939; Smith, 1966). Because the original data were not available for most of these studies, we qualitatively compared them as growth curves.

#### 2.2.2 Comparing within‐species variability of baubella with bacula

Our literature search also uncovered 13 species with quantitative measurements of bacula and baubella length from multiple males and females, all adults. We could therefore compare the coefficients of variation (CV = standard deviation/mean) for bacula and baubella. We tested whether the baubellum CV's differed significantly from baculum CV's using a phylogenetically controlled paired *t* test, as implemented in the phyl.pairedttest function in the R package phytools (Revell, 2012). One of the 13 species, *Parascalops breweri* was represented by *Talpa europaea* on the phylogeny for this test only. The other 12 were already represented in the phylogeny.

In addition to this global approach, we tested whether CV differed between the baubella and the bacula within each species separately, using Feltz and Miller's (1996) asymptotic test for the equality of coefficients of variation, as well as Krishnamoorthy and Lee's (2014) modified signed‐likelihood ratio test. These two approaches were implemented with the functions asymptotic_test2 and mslr_test2, respectively, in the R package cvequality (Marwick & Krishnamoorthy, 2016). We noticed several species where large differences in baubellum CV versus baculum CV failed to produce statistical significance at *p* = .05, and suspected this might be due to small sample sizes available from the literature. To understand the sample size required for statistical significance, we computationally increased sample size until statistical significance was observed.

## 3 RESULTS

### 3.1 Evolutionary patterns

#### 3.1.1 The baubellum shows more evolutionary transitions than the baculum

**Figure 2:**
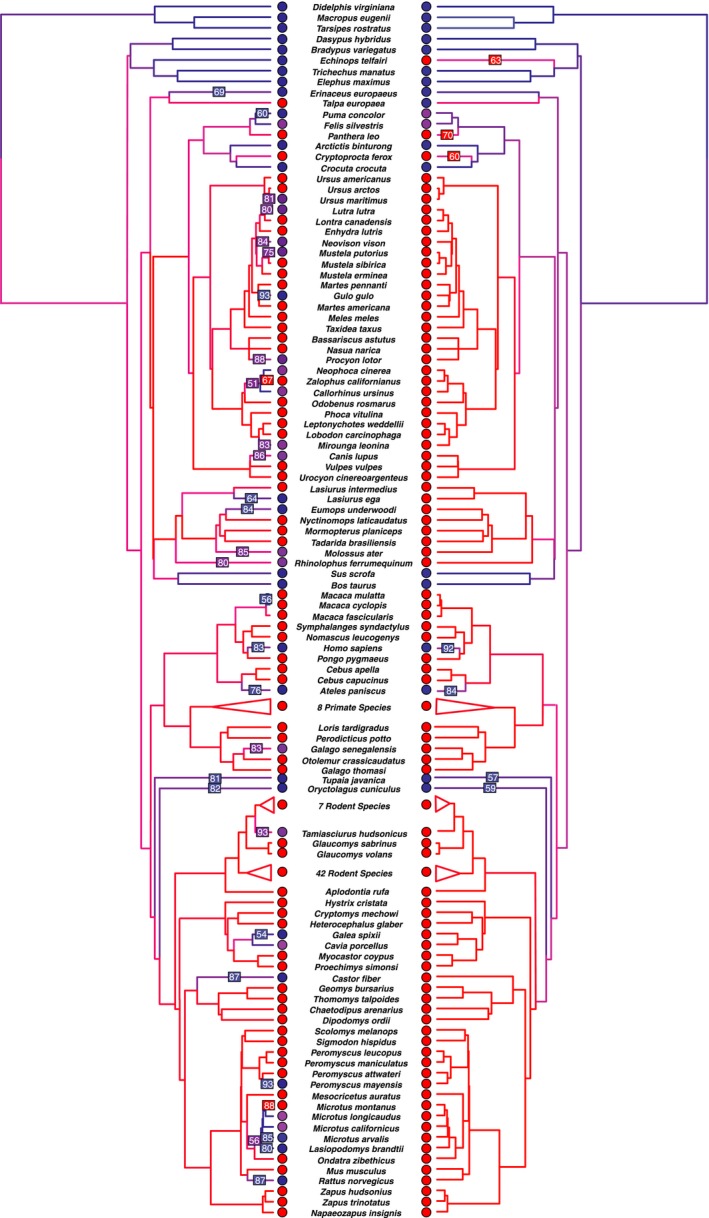
Summary of 1,000 iterations of stochastic mapping for baubellum (left) and baculum (right). Colored circles at terminal nodes indicate character state of each bone: present (red), absent (blue), or polymorphic (purple). Branches are colored according to the average time spent in each state across the 1,000 iterations, on a scale ranging from present (red) through polymorphic (purple) to absent (blue). Boxes on branches indicate “high confidence” character transitions, indicating the percentage of stochastic mapping iterations where transitions occurred on those branches. Boxes on branches are colored according to the state to which the character transitioned (red = present, blue = absent, purple = polymorphic). Note there are more transitions that tend to occur more recently in the baubellum compared to the baculum. A “zoomable” version of this figure is provided in Fig. S1)

A total of 163 species had reliable data for both baculum and baubellum presence and were also represented in a large mammalian phylogeny (Schultz et al., 2016). Of these, 117 had a baubellum, 29 lacked one, and 17 were polymorphic (Figure 2, Table S1). In 134 species, the state of the baubellum matched the state of the baculum (Figure 2, Table S1). However, it should be noted that a large proportion of these (51 of the 134 species) are derived from a single family, Sciuridae (squirrels and chipmunks), so the generality of this pattern should be treated with caution. Sciurid bacula and baubella are regularly used in taxonomy, and so these bones may have been investigated more than in other families (Sutton, 1982, 1995). All 29 species for which states did not match had a baculum but lacked a well‐developed baubellum (either baubellum absent or polymorphic).

**Figure 3:**
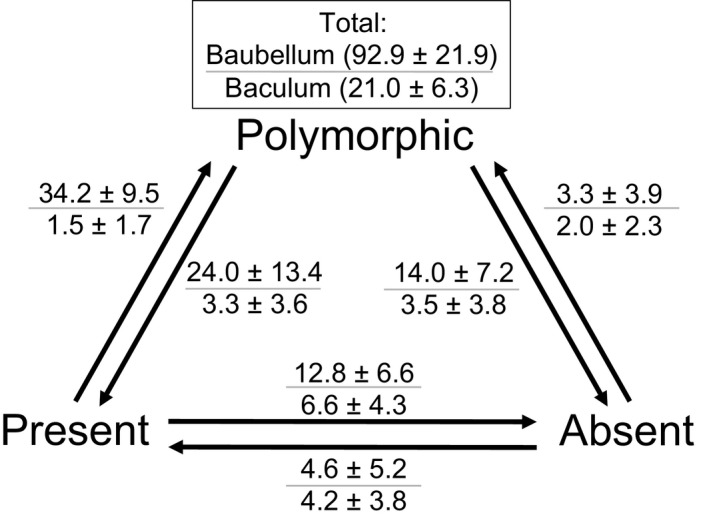
Summary of the average ± standard deviation number of baubellum (number above line) and baculum (number below line) transitions between three states among 1,000 iterations of stochastic mapping. The baculum and baubellum are modeled as three distinct morphological states: present, polymorphic, and absent. Note the baubellum experiences significantly more evolutionary transitions than the baubellum across all transition types (see text)

**Figure 4:**
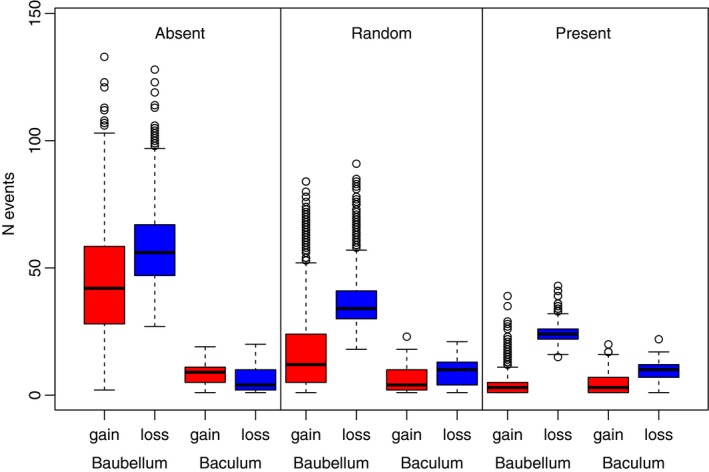
Summary of the number of transitions experienced by the baubellum versus baculum between two different states. Each model recodes polymorphic as present or absent. In all cases, the baubellum experienced significantly more transitions than the baculum (see text)

Under the “polymorphic” model, the baubellum showed significantly more evolutionary transitions compared to the baculum (an average of 92.9 vs. 21.0 transitions, respectively; Wilcoxon Rank Sum Test [WRST] *p* < 10^−15^) (Figures 2 and 3). The other three models also showed significantly more transitions in the baubellum versus the baculum (an average of 102.3 vs. 14.3, 55.1 vs. 14.4, and 28.0 vs. 13.5 baubellum vs. baculum transitions for the “absent,” “random,” and “present” models, respectively, WRST *p* < 10^−15^ in all three cases) (Figure 4). In sum, the baubellum has experienced more evolutionary transitions than the baculum, regardless of how we scored polymorphic species.

In addition, baubellum transitions tended to occur more recently than baculum transitions. For the “polymorphic” model, baubellum transitions occurred an average 27.9 million years ago versus 43.7 million years ago for the baculum. These results held under the other three models (29.3 vs. 39.1, 27.9 vs. 39.2, and 28.2 vs. 41.4 million years ago baubellum vs. baculum transitions for the “absent,” “random,” and “present” models, respectively; LRT, χ^2^ > 997, *df* = 1, *p* < 10^−15^ in all four cases). Therefore, not only has the baubellum experienced more transitions, but those transitions tended to occur more recently than baculum transitions.

#### 3.1.2 Many species with a well‐developed baculum lacked a well‐developed baubellum

Of 145 species with a well‐developed baculum, 12 lacked a well‐developed baubellum (Figure 2, Table S1). These species were widely distributed across the phylogeny and included two primates (Formosan rock macaque, *Macaca cyclopis*; Rhesus macaque, *Macaca mulatta*), six rodents (Maya mouse, *Peromyscus mayensis*; Norway rat, *Rattus norvegicus*; Brandt's vole, *Lasiopodomys brandtii*; Common vole, *Microtus arvalis*; Spix's yellow‐toothed cavy, *Galea spixii*; Eurasian beaver, *Castor fiber*), two bats (Southern yellow bat, *Lasiurus ega*; Underwood's bonneted bat, *Eumops underwoodi*), one carnivore (Wolverine, *Gulo gulo*), and one afrosoricid (Lesser hedgehog tenrec, *Echinops telfairi*). By contrast, there were no species that had a baubellum and lacked a baculum. This could be partially due to study bias, whereby investigators are less likely to look for a baubellum in a species that has no record of a baculum. In addition, eight species were scored as baculum present and baubellum present, but their baubellum remained cartilaginous, unlike the baculum (Table S1).

An additional 17 species had a well‐developed baculum but were polymorphic for the baubellum. These species were also widely distributed, and included one primate (Senegal galago, *Galago senegalensis*) four rodents (Guinea pig, *Cavia porcellus*; California vole, *Microtus californicus*; Long‐tailed vole, *Microtus longicaudus*; American red squirrel, *Tamiasciurus hudsonicus*), ten carnivores (Eurasian otter, *Lutra lutra*; Australian sea lion, *Neophoca cinerea*; North American raccoon, *Procyon lotor*; Northern fur seal, *Callorhinus ursinus*; Domestic dog, *Canis domesticus* [*C. lupus* in phylogeny]; Southern elephant seal, *Mirounga leonina*; European polecat, *Mustela putorius*; Polar bear, *Ursus maritimus*; American mink, *Neovison vison*; Domestic cat, *Felis silvestris*), and two bats (Greater horseshoe bat, *Rhinolophus ferrumequinum*; Black mastiff bat, *Molossus ater*). The percentage of individuals with a baubellum in polymorphic species varied, from one in 100 (1%) of adult female raccoons (Sanderson, 1950), to one in two (50%) in black mastiff bats (Brown, 1967) (Table S1). In sum, many species with a well‐developed baculum lack a well‐developed baubellum, but no species with a baubellum lacked a baculum.

### 3.2 Developmental patterns

#### 3.2.1 The baubellum showed more ontogenetic variation than the baculum

**Figure 5:**
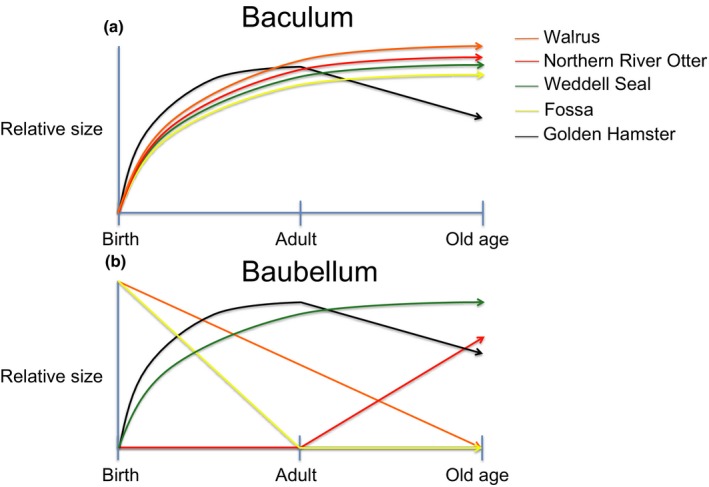
Developmental trajectories of the baculum are consistent across species (top panel), compared to the baubellum in which multiple different paths are observed (bottom panel)

When present, the baculum generally grows steadily from birth to reproductive maturity (Figure 5). The baubellum of two species (Weddell seal, *Leptonychotes weddellii*; Golden hamster, *Mesocritus auratus*) showed similar developmental trajectories (Callery, 1951; Mansfield, 1958; Smith, 1966) (Figure 5). However, three additional species showed striking divergence in developmental patterns (Figure 5). In one species (Northern river otter, *Lontra canadensis*), the baubellum did not begin development until 2 years after birth (Lauhachinda, 1978), in contrast to the male baculum which was present at birth and continued to grow throughout the animal's life (Friley, 1949; Stephenson, 1977). In two species (Walrus, *Odobenus rosmarus*; Fossa, *Cryptoprocta fossa*), baubellum size decreased with age, opposite the developmental patterns of the baculum (Fay, 1955, 1982; Hawkins, 1998; Hawkins et al., 2002).

#### 3.2.2 Within species, the baubellum of adult females is more variable than the baculum of adult males

The baubellum CV's were significantly larger than the baculum CV's across 13 species, as judged by a phylogenetically controlled paired *t* test (*t* = 3.6, *p* = .005).

Across 13 species, 12 had a higher baubellum CV versus baculum CV, seven of which were significantly higher by the asymptotic test and six of which were significantly higher by the modified signed‐likelihood ratio test (Table 1). Some of the nonsignificant results seemed to arise because of small sample size. For example, even though the baubellum of *Spermophilus mexicanus* had a CV more than three times that of the baculum, the difference was not statistically significant, probably because only two females and two males were sampled (Table 1). If we assume existing estimates of CV were reasonably accurate for this species, we would have had to sample at least five males and five females before detecting a significant difference under the asymptotic test (Table 1). The higher baubellum CV was phylogenetically widespread, observed in carnivores, primates, bats, moles, and rodents.

**Table 1:** Comparison of variation in bacula vs. baubella lengths

Species	*N* females	Mean females	*SD* females	CV females	*N* males	Mean males	*SD* males	CV males	Isymptotic statisti	Asymptotic *p* value	mslr statistic	mslr *p* value	Min sample size	References^c^
*Cryptoprocta ferox*	4	5.5	3.3	0.6 ± 0.212	17	72.2	9.6	0.133 ± 0.023	23.76	**.000**	10.12	**.001**	–	Hawkins et al. (2002)
*Lemur catta*	6	0.38	0.06	0.158 ± 0.046	4	1.4	0.14	0.1 ± 0.035	0.65	.419	0.73	.393	21	Drea and Weil (2008)
*Leptonychotes weddellii*	5	35	6.67	0.191 ± 0.06	58	186.31	38.86	0.209 ± 0.019	0.05	.820	0.18	.668	512	Smith (1966)
*Lontra canadensis*	2	10.35	10.11	0.977 ± 0.488	55	94.92	4.46	0.047 ± 0.004	412.61	**.000**	36.17	**.000**	–	Male: Friley (1949); Female: Scheffer (1939)
*Mormopterus planiceps*	6	1.62	0.05	0.031 ± 0.009	9	7.9	0.18	0.023 ± 0.005	0.6	.439	0.42	.517	44	Male: Krutzsch and Crichton (1987); Female: Crichton and Krutzsch (1987)
*Mustela vison*	6^a^	1.02	0.45	0.439 ± 0.127	99	44.6	2.18	0.049 ± 0.003	312.33	**.000**	98.42	**.000**	–	Long and Shirek (1970)
*Parascalops breweri*	2	0.68	0.11	0.157 ± 0.079	2	0.68	0.05	0.072 ± 0.036	0.53	.465	0.36	.549	9	Sinclair (2014)
*Phoca vitulina*	2	7.5	2.12	0.283 ± 0.141	3	127.33	2.52	0.02 ± 0.008	7.81	**.005**	4.4	**.036**	–	Male: Mohr (1962); Female: Scheffer (1949)
*Procyon lotor*	3	13.84	6.58	0.476 ± 0.194	36	102.85	0.06	0.001 ± 0	1237.05	**.000**	204.58	**.000**	–	Long and Frank (1968)
*Sciurus niger*	4	3.38	0.55	0.163 ± 0.058	11	12.36	0.01	0.001 ± 0	83.66	**.000**	68.8	**.000**	–	Long and Frank (1968)
*Spermophilus mexicanus*	2	2.15	0.78	0.362 ± 0.181	2	4.45	0.49	0.111 ± 0.056	1.01	.315	0.76	.385	5	Male: Burt (1960); Female: Layne (1954)
*Taxidea taxus*	4	10.71	1.97	0.184 ± 0.065	2	98.7^b^	9.87^b^	0.1 ± 0.05^b^	0.38	.538	0.52	.471	13	Male: Burt (1960); Female: Long and Frank (1968)
*Urocyon cinereoargenteus*	3	6.33	1.44	0.228 ± 0.093	11	50.94	4.5	0.088 ± 0.019	5.09	**.024**	2.05	.152	–	Male: Long and Frank (1968); Female: Hildebr and (1954)

*N*, number of specimens; *SD*, standard deviation.

CV, coefficient of variation, with standard errors calculated as CV/sqrt(2N). Bold indicates statistical significance at *p *≤ .05.

a 50 females were dissected, baubellum was found in 6.

b Mean taken from Burt 1960, *SD* estimated at 10% of mean based on Long and Frank's (1968) statement that two male bacula were “nearly the same in length”.

c References: unless otherwise indicated, male and female data taken from same study. Full citations can be found in Table S1.

Our finding that the baubellum showed more within‐species variation than the baculum is probably conservative because we based that inference on length measurements that likely underestimated the amount of morphological variation in the baubellum. For example, figure 1 of Long and Shirek (1970) showed a collection of mink baubella that vary dramatically not only in terms of length but also in overall shape, which the present analyses do not capture. In fact, Long and Shirek (1970) remarked of the baubellum that “no other morphological structure known to us has such [high] variation.” In addition, multiple studies have demonstrated the importance of baubellum shape in distinguishing closely related species or subspecies that are otherwise morphologically identical (Adams & Sutton, 1968; Sutton, 1982, 1995). Unfortunately, the existing literature was not detailed enough for us to quantify baubellum variation beyond length measurements.

## 4 DISCUSSION

Sexual dimorphism is common in nature, and the evolutionary and developmental contexts of sexual dimorphism have long‐fascinated biologists (Badyaev, 2002; Darwin, 1874; West‐Eberhard, 2003). A major unsolved question is to what extent sexually dimorphic characters are constrained by the shared genome of males and females (Poissant et al., 2010). Sexual dimorphism is expected to be greatest in species where different optima can be reached via sex‐specific expression of the genome and response to selection. However, most traits are likely to be correlated between sexes, placing significant constraint on the degree to which dimorphism can evolve. At one extreme, a particular state may be beneficial in one sex, but harmful in the other. In the absence of sex‐specific modification of expression, the species will evolve to a phenotypic compromise, where neither sex can reach its optima because of counterselection in the other sex (Poissant et al., 2010). One evolutionary solution to such sexual conflict is sex‐specific expression of the genome, freeing each sex to evolve its own trait value, or even for one sex to lose the trait if it is nonfunctional or deleterious.

Here, we investigate these issues using the baculum and baubellum as a model system, with a focus on testing how strictly the two are correlated. Of 163 species, 134 (83.2%) shared states (both bones present, absent, or polymorphic), which may demonstrate a strong evolutionary correlation (Figure 3). However, investigators may be more likely to look for a baubellum if it is already known that a baculum exists in a species, leading to potential study bias that inflates the correlation of the two states. Nevertheless, the baubellum accumulated more evolutionary transitions than the baculum, and these transitions occurred more recently, demonstrating the two are not strictly correlated. Furthermore, the developmental and morphological variation of the baubellum exceeds that of the baculum. Taken together, our study suggests that baubellum is relatively free to accumulate variation and may not be functional in many lineages.

Other bones, especially “free‐floating” bones like the baculum and baubellum have been gained and lost repeatedly in terrestrial vertebrates, but in almost all cases their presence/absence is perfectly correlated between males and females. For example, mammals have independently lost their clavicles a minimum of four times, and digits in mammals have been independently lost dozens of times (Senter & Moch, 2015). The patella has been independently gained 4–6 times and lost twice in mammals (Samuels et al., 2017), gained multiple times in reptiles (Regnault et al., 2016), and has variable presence in amphibians (Abdala et al., 2017). Even in the face of these multiple independent transitions, clavicles, digits, and patella display the same trait in males and females across species. So far, the intersection of sexual dimorphism and bone losses and gains has only been observed in the mammalian bovids (Family Bovidae) (Caro et al., 2003; Packer, 1983; Stankowich & Caro, 2009). Female and male expression of horns are not perfectly correlated in bovid evolution. Interestingly, similar to our study, there are no known species where females have horns but males do not. Bovid horns are sexually dimorphic in shape, and the most comprehensive analyses conclude they function primarily in males for intrasexual competition and for defense in females (Caro et al., 2003; Stankowich & Caro, 2009). The baculum and baubellum thus represent a highly unusual case of widespread independently evolving sexually dimorphic bones, with the baubellum demonstrating more evolutionary and developmental lability compared to the baculum.

The proximate causes of sexual dimorphism in bacula and baubella appear to be linked to hormonal profiles, or the sensitivity of individuals to various hormones. For example, artificial administration of testosterone in dogs, ferrets, and rats leads to robust development of the baubellum, even though very few female dogs and ferrets, and no female rats, naturally develop one (Baum & Erskine, 1984; Baum et al., 1982; Glucksmann & Cherry, 1972; Kutzler et al., 2012; Murakami & Mizuno, 1984; Shane et al., 1969; Zimbelman & Lauderdale, 1973). Interestingly, castrating males prevented the baculum from reaching an adult stage in multiple species (Howard, 1959; Lyons et al., 1950; Reddi & Prasad, 1967; Sanderson, 1961; Wright, 1950), suggesting androgens are an important mechanistic link between the development of both the baculum and the baubellum. A study that compared skeletal growth in the forepaw and penis in castrated and noncastrated rats concluded that growth factor Somatotropin positively affects bone development in the forepaw but not the penis, and testosterone propionate by contrast affected bone growth in the penis but not the forepaw (Lyons et al., 1950). In the most well‐studied case, inducement of the rat baubellum is time‐dependent and dosage‐dependent and is most effective when administered before 10 days after birth. In laboratory‐raised voles, Ziegler (1961) found that a baubellum‐absent mother had some but not all offspring with baubella; it is possible that natural variation in endogenous androgens or the maternal environment explains such within‐litter variance. The baculum and the baubellum appear to be more androgen‐sensitive than other bones in the skeletal system. If they are more sensitive at the cellular level, these bones would serve as a model for understanding how androgens affect early cell fate decisions in bone development.

Is sexual dimorphism of the baculum and baubellum influenced by the morphology of the penis and clitoris, respectively? The development and evolution of the baubellum cannot be understood without characterization of the soft tissue anatomy in which it resides, namely the female clitoris. However, few studies exist on the comparative anatomy of the clitoris. In one study of 10 species (including primates, moles, and hedgehogs), the internal structure of the clitoris and the baubellum differed greatly not only in size and shape, but also whether they were distal or proximal to the urethra and vaginal opening (Pehrson, 1914). The position of the clitoris varied across 41 eutherian and marsupial species, from deep within the vaginal tract to just inside the vaginal opening or cranial to the vaginal opening (Pavličev & Wagner, 2016). Too few species overlap with our study, therefore it remains unknown how baubellum and clitoral anatomy covary.

In addition to the anatomy of the surrounding soft tissue, behavioral data are required to evaluate whether the baubellum is in fact functional. In many species, the clitoris contains erectile bodies that engorge during copulation (Crichton & Krutzsch, 1987; Drea & Weil, 2008; O'Connell et al., 2005). There is even some speculation that engorgement of the clitoris alters access to the female's reproductive tract, and without it copulation cannot occur (Lönnberg, 1902; Steel, 1885). In males, engorgement of the penis can lead to changes in the orientation of the baculum, probably the result of hydrostatic pressure in the corpora cavernosa pressing against the baculum (Herdina et al., 2015; Kelly, 2000). The stiffening of the corpus cavernosa in the clitoris might have similar effects on the baubellum, which again might shed light on its potential role during copulation. In some primates and pinnipeds, the clitoris and surrounding tissue can undergo changes in color, shape, and/or size during seasonal estrous (Greig et al., 2007; Petter‐Rousseaux, 1962, 1964; Ramaswami & Kumar, 1965). The link to seasonal estrus suggests that the clitoris, and thus the baubellum, may play a role in reproduction in these species. Juvenile female fossa has a very large baubellum and clitoris that gives juvenile females a “masculinized” appearance (Lönnberg, 1902), and it has been speculated that this masculinized phenotype reduces male sexual harassment and female territoriality (Hawkins et al., 2002). Interestingly, female fossa lose their baubellum as they age.

In conclusion, our study demonstrates that the baubellum is relatively free to accumulate evolutionary transitions and developmental variation compared to the baculum. At least in some species, these patterns suggest that the baubellum does not play an important functional role and has become relatively unlinked to the character in males, the baculum. In the future, additional anatomical, behavioral, and developmental data may modify these conclusions in specific cases, but the overall trend appears to be multiple cases of relaxed selection against a general background of developmental and evolutionary correlation. These unusual bones provide a unique model system to understand the evolutionary and developmental mechanisms that give rise to morphological novelty and sexual dimorphism.

## CONFLICT OF INTEREST

None declared.

## AUTHOR CONTRIBUTIONS

MLS, NS, and MD were responsible for the conception of the project, literature review, analysis, and writing of the manuscript. All authors have provided critical review and approved submission of this manuscript.
