# Supplementary material for: The baubellum is more developmentally and evolutionarily labile than the baculum
Source: Ecol Evol. 2017 Dec 20;8(2):1073–83. doi: 10.1002/ece3.3634 (PMC5773289; doi:10.1002/ece3.3634)

Supplementary Figure 1

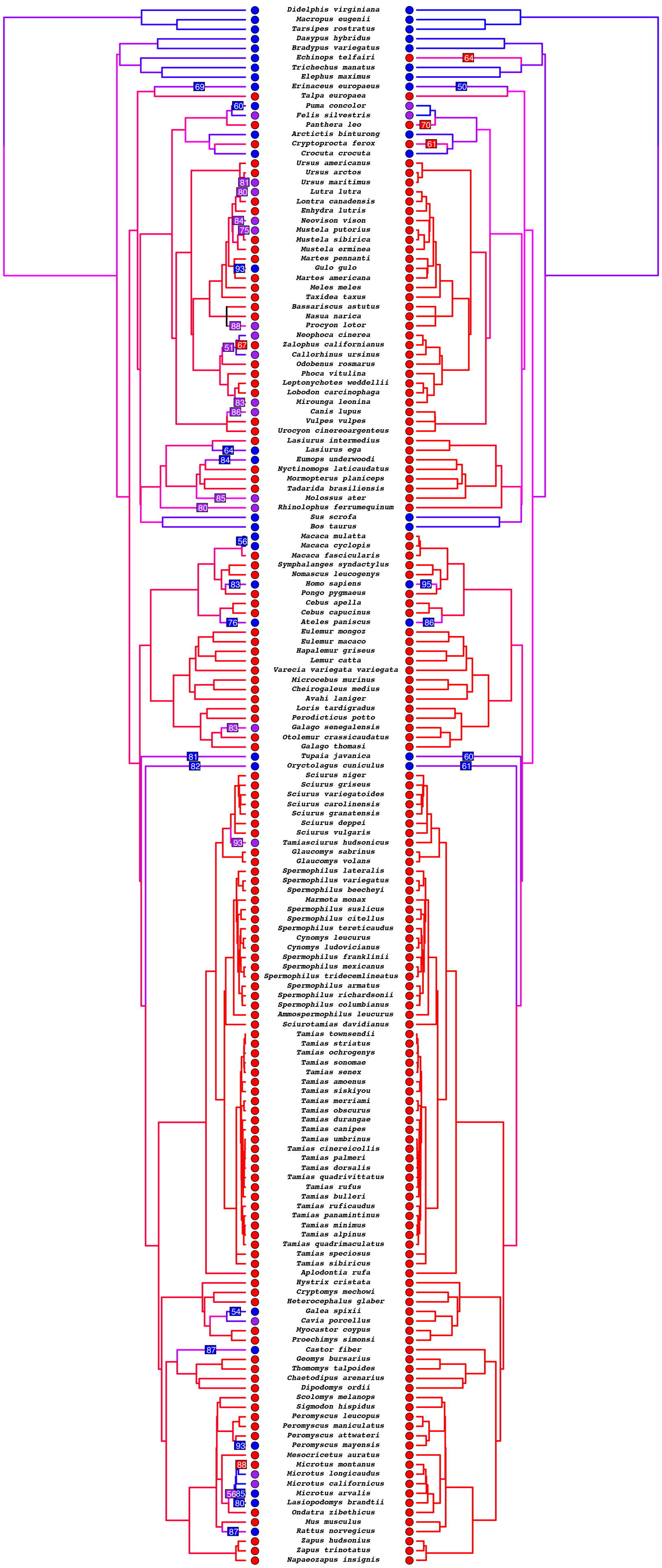

Supplement: Supplementary file 1 [file ECE3-8-1073-s001.pdf]
